# Determinants of domestic violence against women in Ghana

**DOI:** 10.1186/s12889-016-3041-x

**Published:** 2016-05-02

**Authors:** Ebenezer S. Owusu Adjah, Isaac Agbemafle

**Affiliations:** Cyprus International Institute for Environmental and Public Health, Cyprus University of Technology, Limassol, Cyprus; School of Public Health, University of Health and Allied Sciences, Ho, Ghana; School of Medicine, The University of Queensland, Brisbane, Australia

**Keywords:** Domestic violence, Ghana, Woman, Men, Risk factors

## Abstract

**Background:**

The prevalence of domestic violence remains unacceptably high with numerous consequences ranging from psychological to maternal and neonatal mortality and morbidity outcomes in pregnant women. The aim of this study was to identify factors that increased the likelihood of an event of domestic violence as reported by ever married Ghanaian women.

**Methods:**

Data from the 2008 Ghana Demographic and Health Survey (GDHS) was analysed using a multivariate logistic model and risk factors were obtained using the forward selection procedure.

**Results:**

Of the 1524 ever married women in this study, 33.6 % had ever experienced domestic violence. The risk of ever experiencing domestic violence was 35 % for women who reside in urban areas. Risk of domestic violence was 41 % higher for women whose husbands ever experienced their father beating their mother. Women whose mother ever beat their father were three times more likely to experience domestic violence as compared to women whose mother did not beat their father. The risk of ever experiencing domestic violence was 48 % less likely for women whose husbands had higher than secondary education as compared to women whose husbands never had any formal education. Women whose husbands drink alcohol were 2.5 times more likely to experience domestic violence as compared to women whose husbands do not drink alcohol.

**Conclusion:**

Place of residence, alcohol use by husband and family history of violence do increase a woman’s risk of ever experiencing domestic violence. Higher than secondary education acted as a protective buffer against domestic violence. Domestic violence against women is still persistent and greater efforts should be channelled into curtailing it by using a multi-stakeholder approach and enforcing stricter punishments to perpetrators.

## Background

Domestic violence is the intentional use of physical force or power, threatened or actual, against oneself, another person, or against a group or community that either results in or has a high likelihood of resulting in injury or death [1]. According to Act 732 of the parliament of the Republic of Ghana, domestic violence within the context of previous or existing relationship means engaging in acts that constitutes a form of harassment, threat or harm to a person or behaviours likely to result in physical, sexual, economic, emotional, verbal or psychological abuse [2]. Physical abuse is the use of physical force against a person or the deprivation of a person of access to adequate food, water, clothing, shelter, rest, or subjecting a person to inhuman treatment. Sexual abuse refers to the forceful engagement of a person in a sexual contact or a sexual contact by a person aware of having sexually transmitted disease with another person without given the person prior information of the infection. Economic abuse, involves threatened deprivation of financial resources or hindering the use of property in which a person has material interest or is entitled to by law. Emotional, verbal or psychological abuse is any conduct that makes another person feel constantly unhappy, miserable, humiliated, afraid, jittery or worthless.

Domestic violence occurs in all countries but its prevalence varies greatly across the world and even within sub-Saharan Africa [3, 4]. Irrespective of social, economic, religious and cultural groupings, men have been identified as the main perpetrators of domestic violence against women [5]. The prevalence of domestic violence remains unacceptably high with 10–69 % of women worldwide being physically assaulted by an intimate male partner at some point in their lives [6]. Statistics in Ghana indicate that 33–37 % of women have ever experienced domestic violence in the form of intimate partner violence in their relationship [7]. Even in schools, research has shown that 14 and 52 % of girls are victims of sexual abuse and gender-based violence respectively [8]. These estimates may be far less than what actually persists, as violence against women and girls remain a largely hidden problem (sensitive issue) that only few females have the courage to openly confess [9, 10].

There are numerous health consequences of domestic violence particularly against women and children. Some are psychological or emotional in nature and may sometimes result in ill-health [11–14]. For women, physical violence during pregnancy is associated with maternal and neonatal mortality and morbidity [15]. This devastating consequence of violence against women has called for intensified efforts to curtail this ordeal. Existing efforts at the international level include the adoption of the Declaration on the Elimination of Violence Against Women (DEVAW) by the United Nations. Interventions in Ghana include the setting up of Domestic Violence and Victim Support Unit (DOVSU) of the Ghana Police Service, two specialist gender-based violence courts, provision of shelter for survivors of domestic violence and the passage of the Domestic Violence Act 732 on February 21, 2007 [8]. Several studies have pointed out factors associated with domestic violence including but not limited to individual factors (young age, heavy drinking, depression, personality disorders, low academic achievement, low income, witnessing or experiencing violence as a child), relationship factors (marital conflict, marital instability, male dominance in the family, economic stress, poor family functioning), community factors (weak community sanctions against domestic violence, poverty, low social capital), societal factors (traditional gender norms, social norms supportive of violence) [1, 5, 16]. Much of Ghana’s efforts in the fight against domestic violence have been geared towards social, economic and political systems which could be identified as the basic causes of violence against women. This in part is due to the fact that most studies regarding risk factors for domestic violence come from developed countries that have other systems different from those persistent in Ghana as well as other African countries. There is therefore the need to examine these risk factors in the context of the Ghanaian population. The 2008 Ghana Demographic and Health Survey (GDHS) included a series of questions that focused on specific aspects of domestic violence against women. This study seeks to use the 2008 GDHS to identify the underlying and immediate factors associated with domestic violence against women in Ghana to serve as a basis for programme planning and implementation.

## Methods

### Data source

This is a secondary data analysis from the household questionnaire of the 2008 GDHS. A detailed description of the GDHS study design and methods is available elsewhere [7]. Notably, this study was a nationally representative cross-sectional survey that sampled about 12,000 households using a weighted approach. Half of these households were selected for individual interviews and the domestic violence module was administered to women in two-thirds of households selected for the individual interview. Subsequently, only one person was administered the domestic violence module in each selected household. Informed consent was obtained at the beginning of the individual interview and at the beginning of the domestic violence module and additional information was given for domestic violence. Access to demographic and health survey data is managed and provided by MEASURE DHS following an online registration (http://www.dhsprogram.com).

### Study participants

Of the households selected for individual interview, 2,563 women were eligible for the domestic violence module, 17 women were excluded because of lack of privacy, 23 women refused to be interviewed with the domestic violence module and 81 women were not interviewed for other reasons. A total of 2442 (unweighted) women agreed to be interviewed. We excluded never married women as well as participants with missing data (*n* = 765) on covariates included in the multivariable model such as partner’s education level, respondent’s alcohol use, husband’s alcohol use, history of mother beating father and vice versa. This resulted in a sample size of 1524 women for analysis of risk factors for intimate partner violence against ever married women after sampling weight was applied.

### Domestic violence variables

The outcome variable, domestic violence, as defined for this study included violence perpetrated by intimate partners against women and manifested through acts of physical, sexual, and emotional violence. The following seven (7) questions were used to create the variable for physical violence: (Did) your (last) husband/partner ever *i. Slapped you? ii. Twisted your arm or pulled your hair? iii. Pushed you, shook you, or threw something at you? iv. Punched you with his fist or with something that could hurt you? v. Kicked you, dragged you or did beat you up? vi. Tried to choke you or burned you on purpose? vii. Threatened or attacked you with a knife, gun, or any other weapon* [7]*.* A *“yes = 1”* to any of these questions constituted physical violence. If a woman scores from 1 to 7 then physical violence was coded as “1” to represent an event of “physical violence” and if a woman scores “0” then physical violence was coded as “0” to represent an event of “no physical violence”. Furthermore, sexual violence was measured using the following set of questions for women: (Did) your (last) husband/partner ever *i. physically forced you to have sexual intercourse with him even when you did not want to? ii. Forced you to perform any sexual acts you did not want to? *[7]*.* A *“yes = 1”* to either questions constituted sexual violence; as such if a woman gets a score of “1” or “2”, then a code of “1” was assigned to represent an event of “sexual violence”. If a woman scores “0”, then a code of “0” was assigned to represent the event of “no sexual violence”. Subsequently, spousal violence was created as per its definition in the GDHS report by combining physical and sexual violence [7]. Emotional violence was measured in a similar way, using the following set of questions: (Did) your (last) husband ever: *i. Said or did something to humiliate you in front of others? ii. Threatened to hurt or harm you or someone close to you? iii. Insulted you or made you feel bad about yourself?* [7]*.* A *“yes = 1”* to any of these questions constituted emotional violence. Scoring from 1 to 3 was coded as “1” to represent the event “emotional violence”. Otherwise, a code of “0” was assigned to represent the event of “no emotional violence”. The outcome variable, domestic violence was then created as per the definition of domestic violence for this study by combining spousal violence and emotional violence. The event of “no domestic violence” was coded as “0” for participants who did not experience either spousal or emotional violence. For those who experienced only emotional violence, only spousal violence and both spousal and emotional violence, a code of “1” was assigned to represent the event of “ever experienced domestic violence”. Covariates considered as risk factors were selected on the basis of causal assumption derived from subject matter knowledge. These included age of respondent, place of residence, educational level of respondent and partner, religion, wealth index, marital status, employment status of both responded and partner and alcohol use by both respondent and partner [1, 4, 14, 17].

### Analysis

Distribution of categorical variables were reported as frequency counts whilst associations were tested using chi-square or fisher’s exact test. Univariate logistic regression analysis was initially performed to evaluate the ability of each covariate to predict the event “ever experienced domestic violence”. Predictors with some degree of association from the univariate analyses (*p* < 0.25) were entered into a preliminary multivariate logistic model [18] either as continuous variables or categorized as quartiles and those that showed some degree of association (*p* < 0.25) were added one by one until no remaining variable produces a significant *F* statistic (forward selection). The forward selection model was chosen over simultaneous model as this study was designed to select from a group of independent variables, the one variable at each stage which makes the largest contribution to R^2^. To ensure that the predictor variables included in the model were independent of each other, variance inflation factor was used as a measure colinearity and none of the predictor variables in final model was highly associated with each other. Data were analysed using SAS version 9.2 (SAS Institute) and all statistical tests were two tailed and a *p* < 0.05 was considered statistically significant.

## Results

### Socio-demographic characteristics

**Fig. 1:**
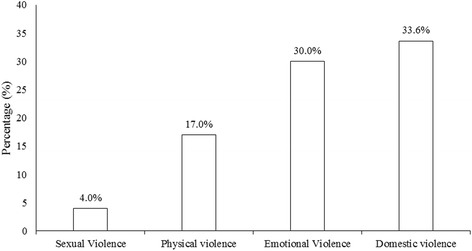
Forms of domestic violence against women in Ghana

**Fig. 2:**
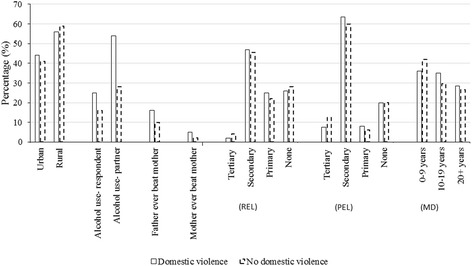
Distribution of potential risk factors by domestic violence (legend: REL = respondents education level, PEL = Partner education level, MD = marital duration)

Of the 1524 ever married women in this study, 33.6 % had ever experienced domestic violence (some form of sexual, physical or emotional violence) and 87 % were currently married. The most frequently reported violence against women in Ghana was emotional violence, followed by physical and sexual violence in that order (Fig. 1). The median age of the women in this study was 33 years and the majority (87 %) were currently married (Table 1). Educational level was higher for men (spouse/partner) than women as shown in Table 1. Seventy-six percent (76 %) of the women were Christians and 89 % also engaged in some form of employment. About 40 % of the women were in the lowest quintile of the wealth index. The proportion of women (19 %) who consumed alcohol was less than the number of men who drank alcohol (37 %; Table 1). Fewer women (3.0 %) reported witnessing mother ever beat father as compared to 12.1 % who mentioned that their father ever beat their mother. For about half of the women, the average number of children reported ranged from one to three. The most common duration of marriage as reported by 40 % of the women was 0–9 years (Table 1). There were no differences in proportion for place of residence, educational level, and marital duration between the women who had ever experienced domestic violence as compared to those who had never experienced it (Fig. 2). However, there were differences in alcohol use and family history of violence; with the proportion skewed towards the women who had ever experienced domestic violence as compared to those who had never experienced it (Fig. 2).

**Table 1:** Comparison of socio-demographic characteristics of study participants

Characteristics	*n* (%)
Age (Median, IQR)	33 (16–49)
Place of residence (n, %)	
Urban	639 (42.0)
Rural	885 (58.0)
Educational level-respondent (n, %)	
None	420 (27.6)
Primary	348 (22.8)
Secondary	700 (46.0)
Higher than secondary (Tertiary)	56 (3.6)
Educational level-partner (n, %)	
None	308 (20.2)
Primary	112 (7.3)
Secondary	932 (61.2)
Higher than secondary (Tertiary)	172 (11.3)
Religion (n, %)	
Christian	1167 (76.6)
Muslim	227 (14.9)
Traditional	73 (4.8)
No religion	54 (3.5)
Other	3 (0.2)
Respondent currently employed (n, %)	
Yes	1358 (89.1)
No	166 (10.9)
Partner currently employed (n, %)	
Yes	1519 (99.6)
No	5 (0.4)
Husband drinks alcohol (n, %)	
Yes	568 (37.3)
No	956 (62.7)
Respondent drinks alcohol (n, %)	
Yes	294 (19.3)
No	1230 (80.7)
Marital status (n, %)	
Currently married	1326 (87.0)
Formerly married	198 (13.0)
Wealth index (n, %)	
Poorest	288 (18.9)
Poorer	309 (20.3)
Middle	294 (19.3)
Richer	328 (21.5)
Richest	305 (20.0)
Mother ever beat father (n, %)	
Yes	46 (3.0)
No	1478 (97.0)
Father ever beat mother (n, %)	
Yes	185 (12.1)
No	1339 (87.9)
First intercourse (n, %)	
Forced	168 (11.0)
Wanted	1356 (89.0)
Total children ever born (n, %)	
None	112 (7.4)
1–3	779 (51.1)
4–6	486 (30.7)
7+	165 (10.8)
Marital duration (n, %)	
0–9 years	609 (39.9)
10–19 years	496 (32.6)
20+ years	419 (27.5)

### Risk factors

Higher than secondary level of education of partner from the univariate analysis independently lowered a woman’s risk of domestic violence by 45 % whilst women who had higher than secondary education were 55 % less likely to experience domestic violence (*p*-value < 0.05; Table 2). Alcohol use by women was independently associated with a 71 % (95 % confidence interval (CI) = 1.34–2.17) increased likelihood of experiencing domestic violence of whereas alcohol use by partner or husband increases the same likelihood by about 3 fold (OR = 2.55, 95 % CI = 2.07–3.15; Table 2). History of mother ever beating father independently increases risk of domestic violence by 4folds whilst history of father ever beat mother was associated with a 92 % chance of domestic violence (*p*-value < 0.0001). Age, employment status and wealth index were not independent risk factors for domestic violence as shown in the univariate analysis in Table 2.

**Table 2:** Odds ratios of risk factors for domestic violence from univariate analysis

Variables	OR (95 % CI)	*p*-value
Age	1.00 (0.99–1.02)	0.4628
Total children born	1.04 (0.99–1.08)	0.0940^a^
Place of residence		
Urban	1.14 (0.93–1.40)	0.2176 ^a^
Rural	Reference	
Educational level-respondent		
Tertiary	0.45 (0.22–0.90)	0.0252^a^
Secondary	1.11 (0.87–1.41)	0.0739^a^
Primary	1.29 (0.98–1.70)	0.3933
No education	Reference	
Educational level-partner		
Tertiary	0.55 (0.22–0.83)	0.0039^a^
Secondary	1.00 (0.78–1.27)	0.7287
Primary	1.08 (0.71–1.63)	0.9710
No education	Reference	
Respondent currently employed		
Yes	1.10 (0.79–1.53)	0.5726
No	Reference	
Partner currently employed		
Yes	1.56 (0.31–7.74)	0.5894
No	Reference	
Respondent drinks alcohol		
Yes	1.71 (1.34–2.17)	<0.0001^a^
No	Reference	
Husband/partner drinks alcohol		
Yes	2.55 (2.07–3.15)	<0.0001^a^
No	Reference	
Religion		
Muslim	1.18 (0.90–1.54)	0.2442^a^
Traditional	0.96 (0.62–1.48)	0.8397
No religion	0.92 (0.54–1.57)	0.7583
Other	0.66 (0.07–6.36)	0.7170
Christian	Reference	
Respondents mother beat father		
Yes	4.05 (2.24–7.31)	<0.0001^a^
No	Reference	
Respondents father beat mother		
Yes	1.92 (1.43–2.57)	<0.0001^a^
No	Reference	
Wealth index		
Richest	0.90 (0.65–1.23)	0.5077
Richer	1.10 (0.81–1.48)	0.8397
Middle	0.94 (0.54–1.57)	0.7583
Poorer	0.85 (0.63–1.16)	0.7170
Poorest	Reference	
Last intercourse		
4+ weeks	1.06 (0.85–1.32)	0.6011
3 weeks	0.65 (0.37–1.34)	0.1285^a^
2 weeks	1.13 (0.80–1.60)	0.4944
1 week	Reference	
Marital duration		
20 + years	1.17 (0.90–1.52)	0.2344^a^
10–19 years	1.25 (0.99–1.58)	0.0626^a^
0–9 years	Reference	

^a^entered into the multivariable logistic regression

After adjusting for other potential risk factors (age, total number of children, employment status, religion, wealth index, last intercourse and marital duration), place of residence, educational level (respondent or partner), husband alcohol consumption and father/mother ever beating spouse were significant predictors of domestic violence among women. Women who reside in urban areas were at 35 % increased risk of ever experiencing domestic violence as opposed to women in rural areas. Educational level seems to confer a protective effect against domestic violence. The higher the educational level of partner, the lower a woman’s risk of ever experiencing domestic violence. The risk of ever experiencing domestic violence was 48 % lesser for women whose husbands had higher than secondary education as compared to women whose husband never attended school (Table 3). The odds ratio for experiencing domestic violence for women whose husbands consume alcohol was 2.52 as shown in Table 3. This indicates that women whose husbands drink alcohol were 2.5times more likely to experience domestic violence. This effect of alcohol use remained statistically significant given that educational level, place of settlement and father/mother ever beat partner were included in the multivariate model. The results of this study also revealed that prior family history of domestic violence is a strong predictor for ever experiencing domestic violence in later life. Notably, women whose mother ever beat father were three times more likely to experience domestic violence as compared to women without family history of domestic violence. The odds ratio for women whose father ever beat mother was 1.41 indicating that the risk of ever experiencing domestic violence for those women was 41 % higher compared to women whose father never beat their mother.

**Table 3:** Risk factors for domestic violence in Ghana

Risk factors	OR (95 % CI)	*p*-value
Place of residence		
Urban	1.35 (1.08–1.70)	0.0098
Rural	Reference	
Educational level-partner		
Higher	0.52 (0.34–0.80)	0.0032
Secondary	0.96 (0.74–1.25)	0.8626
Primary	1.04 (0.67–1.60)	0.7731
No education	Reference	
Husband drinks alcohol		
Yes	2.52 (2.04–3.12)	<0.0001
No	Reference	
Respondents mother beat father		
Yes	3.04 (1.61–5.76)	0.0006
No	Reference	
Respondents father beat mother		
Yes	1.41 (1.02–1.96)	0.0401
No	Reference	

OR’s were adjusted for age, total number of children, employment status, religion, wealth index, last intercourse and marital duration

## Discussion

Factors associated with domestic violence that have previously been documented were mostly from countries in Asia and Latin America with varying political, economic and cultural differences and very little focus on sub-Saharan Africa [5, 9, 12, 19–21]. These factors reported in other countries may not necessarily lead to an increase in the likelihood of a Ghanaian woman’s risk of domestic violence. The aim of this study was therefore to identify specific factors that increased the likelihood of an event of domestic violence as reported by a representative sample of married Ghanaian women. To the best of our knowledge, this study is one of the first to evaluate risk factors of domestic violence in Ghana. In this study, physical and sexual violence were less reported by the women as compared to emotional violence. In Ghana as compared to Bangladesh [14], emotional violence was reported as the most common form of domestic violence against women. This difference might be attributable to methodological complexities and sociocultural variations among women in these countries. Although physical and sexual violence were more readily quantifiable than emotional abuse as reported in Bangladesh [14], results of qualitative research demonstrated that emotionally-abusive acts might be more devastating [4]. However, the issue of emotional violence is complex, and more data are needed to understand its complexities.

In this study, the women were not only exposed to various forms of abuse but were more likely to experience an event of domestic violence if they lived in the urban areas compared to living in rural areas. This is because, most of the women in urban areas may reside in slums or poor urban areas and/or may have higher wealth index (economic status) which may increase their risk of domestic violence. A previous study in India reported high prevalence of domestic violence among women living in slums [22]. Also, Counts et al*.* [23] reported that in cities where women have a higher economic status, they were seen as having sufficient power to change traditional gender roles; it is at this point that domestic violence is at its highest.

There was a positive association between past exposures to violence in terms of father abusing mother or vice versa and a woman’s current status of ever experiencing domestic violence. Notably, there were differences in family history of domestic violence exposure risk as reported by women in this study compared to that reported by men in North India [16]. This may be attributed to bias as men who may be the main aggressors [1] are more likely to under report events that are defined as physical, sexual or emotional violence whereas women are more likely to over report them. This bias may be of interest to other researchers working on domestic violence or any form of violence. History of family violence has been linked to domestic violence in later life and studies have shown that exposure to violence affects children’s aptitude and perpetuates the intergenerational transmission of violence [1, 16, 24–26]. Our study does not provide information about the mechanism through which family history of violence exerts their effect on a child in later life, but it seems reasonable to assume that at least part of the effect is through increased or sustained occurrence of such events such that the attitude of accepting violence in marriage becomes a norm.

The likelihood of domestic violence occurrence was common among women who reported that their partner drinks alcohol. This supports evidence from previous studies [16, 19, 20, 22, 27] perhaps making alcohol use the most common risk factor of domestic violence against women. Many researchers believe that alcohol operates as a situational factor, increasing the likelihood of violence by reducing inhibitions, clouding judgement and impairing an individual’s ability to interpret cues [27, 28].

Regular alcohol consumption by other partner, exposure to harsh physical discipline during childhood and witnessing father beating the mother during childhood have emerged as risk factors of domestic violence, all of which put women at an increased risk of depression, suicide attempts, psychosomatic disorders and physical injury [1, 12]. The consequences and costs of domestic violence may have impact at the individual, family, community and national level. Costs due to domestic violence may include healthcare (mental and physical) costs to the survivor and her family, employment and financial difficulties and the effects on children. Children who witness domestic violence are more likely to have emotional and behavioural problems, perform poorly in school and be at risk of perpetrating or experiencing domestic violence in later life [17]. Violence against women may have undermined efforts to realize the Millennium Development Goals (MDGs) as it hinders poverty reduction efforts and has inter-generational consequences. It also undermines women’s ability to exercise their reproductive rights with grave consequences for maternal and child health.

In keeping with previous findings, partner education particularly higher than secondary education of husband or partner offers a protective effect against domestic violence. This is in agreement with a recent review of data from 17 sub-Saharan countries that reported that intimate partner violence against women was more acceptable amongst the less educated [26]. The present study revealed that educational level of partners was slightly higher than that of the women. A study in New Zealand demonstrated that low academic achievement was one of the risk factors predicting physical abuse of partners by men [29]. Interestingly, a study in India reported higher than secondary level of education of both the woman and her partner as a protective buffer, suggesting the importance education could play in reducing violence against women [12, 30]. Women and/or partners with higher than secondary education may be less likely to be abused or abuse their partners because they perceive each other as valuable and perhaps more valuable by their extended families [30]. Although domestic violence reported was quite high, 87 % of the women were currently married, a finding consistent with a study in Tanzania [30]. According to McCloskey et al. [30], majority of women in Tanzania with an intimate partner violence history still live with their violent partners although higher wealth index and education may give women more power to leave their abusive partners, emphasising the value women in Africa place on unions.

The cross-sectional nature of the data limits ability to draw casual inferences. Also, all assessments were based on self-reports by respondents, and are likely to be gross underestimates or overestimates which can undermine the true prevalence of domestic violence in Ghana. Despite these limitations, data for this study comes from a large nationally representative survey and variables from individual, relationship, community and societal levels were tested. Also, this study has provided valuable data on risk factors for domestic violence and established domestic violence as one of the major public health problems in Ghana.

## Conclusion

Place of residence, alcohol use by husband and family history of violence do increase a woman’s risk of domestic violence. Higher than secondary education acted as a protective buffer against domestic violence. Domestic violence remains unacceptably high in Ghana and should be treated as one of the major public health problems that needs a multi-stakeholder approach based on culturally acceptable and sustainable intervention strategies to deal with it.

### Ethics approval and consent to participate

Not applicable.

### Consent for publication

Not applicable.

### Availability of data and materials

Access to demographic and health survey data is managed and provided by MEASURE DHS following an online registration (http://www.dhsprogram.com).

## Abbreviations

DEVAW: Declaration on the Elimination of Violence Against Women
DHS: Demographic and Health Survey
DOVSU: Domestic Violence Support Unit
GDHS: Ghana Demographic and Health Survey
